# Origin of Subgap States in Normal-Insulator-Superconductor van der Waals Heterostructures

**DOI:** 10.1021/acs.nanolett.2c02777

**Published:** 2023-03-16

**Authors:** Paritosh Karnatak, Zarina Mingazheva, Kenji Watanabe, Takashi Taniguchi, Helmuth Berger, László Forró, Christian Schönenberger

**Affiliations:** ^†^Department of Physics and ^#^Swiss Nanoscience Institute, University of Basel, CH-4056 Basel, Switzerland; ‡ Research Center for Functional Materials, National Institute for Material Science, 1-1 Namiki, Tsukuba 305-0044, Japan; § International Center for Materials Nanoarchitectonics, National Institute for Materials Science, 1-1 Namiki, Tsukuba 305-0044, Japan; ∥ Institute of Condensed Matter Physics, Ecole Polytechnique Fédérale de Lausanne, CH-1015 Lausanne, Switzerland; ⊥ Stavropoulos Center for Complex Quantum Matter, Department of Physics, University of Notre Dame, Notre Dame, Indiana 46556, United States

**Keywords:** NbSe_2_, superconductivity, nanostructures, Andreev bound state, subgap excitation, tunneling

## Abstract

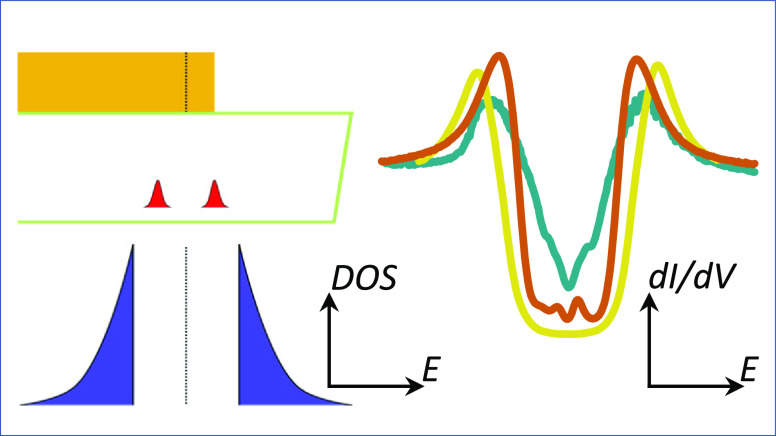

Superconductivity in van der Waals materials, such as NbSe_2_ and TaS_2_, is fundamentally novel due to the effects of dimensionality, crystal symmetries, and strong spin–orbit coupling. In this work, we perform tunnel spectroscopy on NbSe_2_ by utilizing MoS_2_ or hexagonal boron nitride (hBN) as a tunnel barrier. We observe subgap excitations and probe their origin by studying various heterostructure designs. We show that the edge of NbSe_2_ hosts many defect states, which strongly couple to the superconductor and form Andreev bound states. Furthermore, by isolating the NbSe_2_ edge we show that the subgap states are ubiquitous in MoS_2_ tunnel barriers but absent in hBN tunnel barriers, suggesting defects in MoS_2_ as their origin. Their magnetic nature reveals a singlet- or a doublet-type ground state, and based on nearly vanishing *g* factors or avoided crossings of subgap excitations, we highlight the role of strong spin–orbit coupling.

Superconductivity in the two-dimensional limit is driven by a unique interplay among dimensionality, crystal symmetries, correlated electron effects, and, if present, the role of spin–orbit coupling. This often results in various competing ground states and gives rise to rich novel phenomena. Ultimately, two-dimensional van der Waals superconductors are illustrative examples. Naturally superconducting NbSe_2_ and TaS_2_ have been recently isolated and studied,^1,2^ and MoS_2_ has been doped into a superconducting state.^3^ In their monolayer or few-layer forms, these van der Waals superconductors display novel phenomena, such as the survival of superconductivity up to tens of teslas of applied in-plane magnetic field,^1,2^ layer -dependent superconducting properties,^2^ and competition with other phases.^4^ Furthermore, it is predicted that these materials can be externally tuned to host novel topological phases,^5,6^ and there are expectations of the presence of unconventional pairing mechanisms in Ising superconductors.^7^

These features essentially result from the large spin–orbit coupling (SOC) and the crystal symmetry in these materials. For this SOC, called the Ising type, the corresponding spin orbit magnetic field points out of plane and in opposite directions in the opposite valleys of the hexagonal Brillouin zone of these materials.^1,3^ This splits the spin-degenerate bands, and the majority singlet Cooper pairs are expected to be formed from opposite valleys. As the large spin orbit magnetic field (some estimates indicate *B*_so_ ≈ 100 T^3^) pins the spins out of plane, an applied in-plane magnetic field (usually smaller than *B*_so_) hardly affects the electron spins and thus the Cooper pairs survive large Zeeman fields.

Recently, proximity-induced superconductivity in semiconducting nanostructures has been widely investigated,^8−12^ primarily driven by the proposals for topological quantum computation.^13,14^ Additionally, low-dimensional structures coupled to van der Waals superconductors with a large SOC provide a rich platform for investigating the nature of Andreev bound states. They may also offer insights into the unconventional superconducting properties. In this regard, tunnel spectroscopy is a versatile tool for probing the superconducting density of states (DOS). Electronically gapped van der Waals materials provide high-quality tunnel barriers that allow unprecedented control over the barrier thickness and the interface quality. They are also especially well suited to probing the air-sensitive van der Waals superconductors.^15−17^ Tunnel spectroscopy in such heterostructures has revealed the presence of Andreev levels in the subgap spectrum.^17,18^ However, the exact origin and nature of these bound states have not been systematically investigated, and it is not known if such bound states reside in the tunnel barriers or are hosted on the NbSe_2_ surface.^19−21^ The role of spin–orbit coupling in determining the Andreev-level ground state and their magnetic nature also remains to be understood.

In this work we perform tunneling spectroscopy on NbSe_2_ by utilizing MoS_2_ or hexagonal boron nitride (hBN)^22^ as a tunnel barrier and Ti/Au as the normal leads. We find that the single-particle gapped spectrum is often interrupted by the presence of subgap excitations, and we probe their origin by studying various heterostructure designs. We show that the edge of NbSe_2_ hosts many defect states, some of which are strongly coupled to the superconductor. However, we also observe subgap excitations in devices where the NbSe_2_ edge is electrically isolated. We show that these subgap excitations arise from defects in MoS_2_ and are absent in hBN tunnel barriers. We probe the magnetic nature of these subgap states by studying their evolution in applied magnetic fields and reveal the nature of ground states as well as highlight the role of spin–orbit coupling.

The normal-insulator-superconductor (NIS)-type planar tunnel junctions are fabricated by stacking MoS_2_ (three to five layers) or hBN (three layers) on NbSe_2_ crystals (∼3–20 nm) in a glovebox in an N_2_ atmosphere. MoS_2_ or hBN acts as the tunnel barrier and prevents NbSe_2_ from oxidation; see the schematic and a representative device image in Figure 1a. We have studied 8 devices and more than 50 tunnel junctions, and a summary of results is presented here. Further details of fabrication, device parameters, and measurements can be found in the Supporting Information (SI).

**Figure 1:**
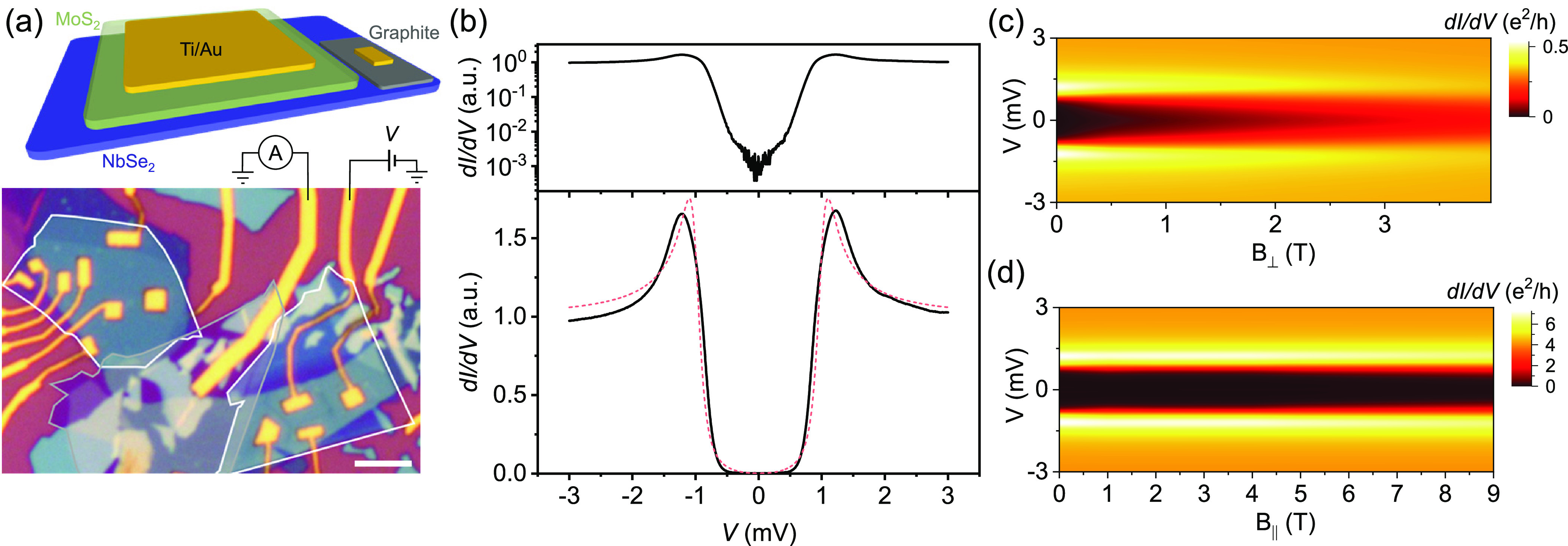
Device design for planar tunneling devices and differential conductance measurements. (a) Schematic shows the normal-insulator-superconductor junctions formed by depositing Ti/Au on the MoS_2_ (or hBN) and NbSe_2_ stack. The optical image shows a typical device with MoS_2_ (white outline) and graphite (gray outline) transferred on NbSe_2_ crystal (bluish color). The scale bar is 5 μm. (b) Measured d*I*/d*V* shows a hard superconducting gap with a suppression factor of *G*_N_/*G*_0_ ≈ 800. The dashed red curve is eq 1 with the parameters Δ ≈ 1.0 meV, Γ ≈ 0.11 meV, and *T* = 255 mK. (c) Out-of-plane magnetic field leads to the softening of the superconducting gap. (d) d*I*/d*V* measured in an in-plane magnetic field shows that the superconducting gap is robust up to 9 T.

The differential conductance across an NIS junction can be written as^23,24^
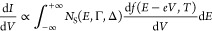
1where *N*_S_ is the single-particle DOS as a function of energy *E* for the superconducting electrode with a superconducting gap Δ and a broadening parameter Γ; *f*(*E*, *T*) is the Fermi–Dirac distribution at a finite temperature *T*; and *V* is the bias voltage applied across the tunnel barrier. The superconducting DOS can be modeled by the Dynes formula^25^
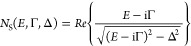
2

It is instructive to see that at zero temperature eq 1 reduces to d*I*/d*V* ∝ *N*_S_(*eV*, Γ, Δ) and the differential conductance measurement across a tunnel barrier probes the DOS of the superconductor. At finite temperature, the DOS features are broadened by ∼*k*_B_*T*. One such measurement is shown in Figure 1b, with a well-defined superconducting gap and a suppression factor *G*_N_/*G*_0_ ≳ 800, where *G*_0_ and *G*_N_ are the differential conductance in the superconducting gap (typically at *V* = 0 or the minimum conductance in the gap) and outside the gap (typically *V* ≈ 3 mV), respectively, as emphasized on the log scale in the top panel of Figure 1b. We typically observe hard gaps across our tunnel barriers with *G*_N_/*G*_0_ ≳ 100, indicating high-quality tunnel barriers and consequently the suppression of Andreev processes. A plot of eq 1 with a gap of Δ ≈ 1.0 meV and broadening parameter Γ ≈ 0.11 meV is shown in Figure 1b. (See the Supporting Information for further discussion on gap fitting.) Moreover, d*I*/d*V* measurements performed in a perpendicular magnetic field reveal significant softening of the superconducting gap (Figure 1c), as expected for NbSe_2_ at this scale due to the orbital depairing.^26,27^ However, the Ising protection against an applied in-plane magnetic field is noticeable for NbSe_2_, as seen in Figure 1d. We observe that the superconducting gap is robust (*G*_N_/*G*_0_ ≈ 100) up to 9 T, limited only by the cryostat magnet. This allows us to study the behavior of the subgap states in a large (in-plane) magnetic field, as discussed later.

Unlike the spectrum shown in Figure 1b, however, we often observe discrete subgap features in MoS_2_ tunnel barrier junctions (Figure 2a). Such subgap features can result from discrete electronic states in the tunnel path, modified by the superconducting proximity effect. The discrete states themselves may arise from a defect or an impurity in the tunnel barrier or on the surface of the superconductor.^19−21^ The formation of such Andreev levels has recently been widely explored, especially in semiconducting nanowires coupled to superconductors, and is fairly well understood.^9,11,12^ We model the defect state as a quantum dot coupled to a superconductor. A spin-degenerate single orbital level in an isolated quantum dot has four eigenstates |0⟩, |↑⟩, |↓⟩, and |↑↓⟩. When the quantum dot couples to a superconductor, the empty |0⟩ and the doubly occupied states |↑↓⟩ are hybridized via virtual Andreev processes that exchange two electrons with the dot, as shown in the schematic in Figure 2b. If the quasiparticles in the superconductor can be neglected (Δ → *∞*, the so-called superconducting atomic limit), then this hybridization results in two BCS-like singlet eigenstates  and , given by the Bogoliubov–de Gennes (BdG) transformation,^28−30^ where *u* and *v* are the BdG amplitudes. For single average occupancy of the dot, the system has two possible ground states: either the degenerate doublet |↑⟩, |↓⟩ or the singlet eigenstate . The energy of the singlet states is given by^29^
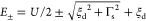
where *U* is the charging energy, ξ_d_ = ϵ_d_ + *U*/2 with ϵ_d_ being the bare energy of the doublet states {|↑⟩, |↓⟩}, and Γ_s_ is the coupling to the superconductor. Therefore, the competition between the ground states {|↑⟩, |↓⟩} and  depends on the relative magnitudes of various energy scales in the system. In general, a stronger coupling Γ_s_ to the superconductor favors a singlet  ground state whereas a large charging energy *U* results in a doublet ground state {|↑⟩, |↓⟩}. In this work, we discuss the subgap features in terms of the Andreev bound states, and this framework holds when the quasiparticles in the superconductor do not play a role. However, in principle our experiments cannot distinguish if the singlet is the superposition of |0⟩ and |↑↓⟩ (Andreev bound state) or is formed between one electron on the dot and another on the superconductor (Yu–Shiba–Rushinov state).^31−33^ Quasiparticles in the superconductor could play a role if Δ ≈ Γ_s_.

**Figure 2:**
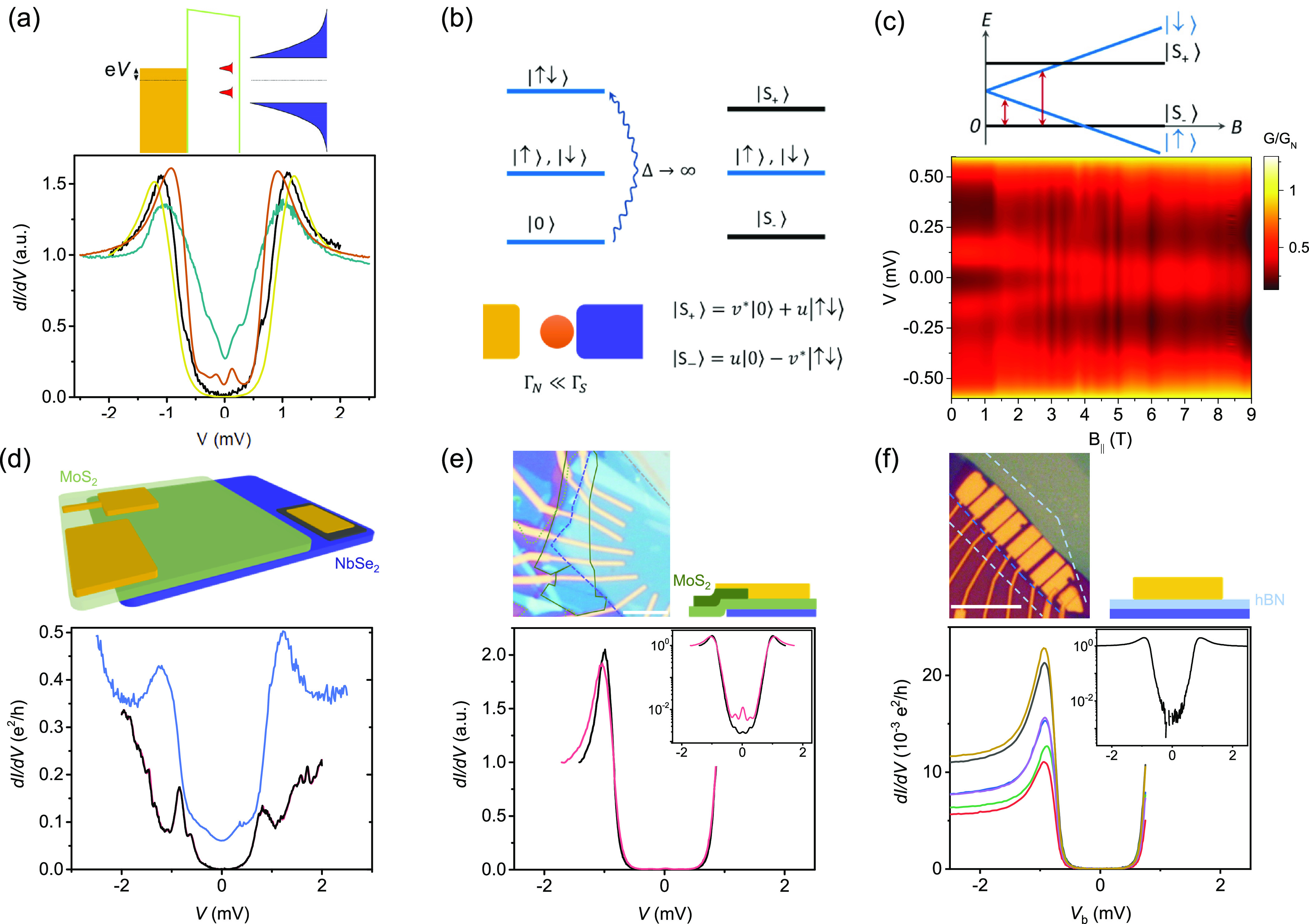
Origin of subgap states. (a) d*I*/d*V* measurements show the presence of subgap excitations. Schematic shows a possible mechanism where a defect strongly couples to the superconductor. Normalized d*I*/d*V* is shown for four different junctions in device D10. (b) Electronic states in a quantum dot are modified when it is strongly coupled to the superconductor. (c) Magnetic field evolution of the subgap excitations indicates that at *B* = 0 the ground state is a singlet but the system undergoes a quantum phase transition to a doublet ground state at a finite magnetic field. Red arrows denote the excitation energies in the schematic. The jumps in magnetic field are a result of the imperfect alignment of *B*_∥_ that leads to discrete units of flux entering the tunnel junction area. (d) d*I*/d*V* measurements on the edge of NbSe_2_. Repeated runs are shown by black and red lines (barely visible, lie on top of each other), while another edge contact is shown in blue. (e) d*I*/d*V* measurements with the edge of NbSe_2_ electrically isolated by using a thicker MoS_2_ (solid green outline) at the edge of NbSe_2_ (blue dashed line). Scale bar is 5 μm. The inset on a log scale highlights the presence of subgap excitations. (f) d*I*/d*V* measurements for hBN used as a tunnel barrier show the absence of subgap excitations. The inset on a log scale shows the absence of subgap excitation down to the measurement noise floor. The scale bar in the optical image is 10 μm.

Applying a dc voltage bias *V* across the tunnel barrier, which is equivalent to the energy of the excited state, results in the transfer of a single electron into the dot (with *N* electrons) from the normal lead. This electron can form a Cooper pair to enter the superconductor; consequently, a hole is retro-reflected into the normal lead. Symmetric across the Fermi energy, a time-reversed process occurs and can be observed as a similar feature at the opposite dc voltage bias. Thus, the electron–hole symmetric subgap features in tunnel spectroscopy probe the excitation energy of the subgap states (*N* to *N* ± 1 transitions) in the lowest order. An external magnetic field causes Zeeman splitting of the spin-degenerate doublet states and provides a key tool to study the nature of the localized ground states. See Figure 2c and the discussion later.

We first investigate the origin of such subgap excitations: whether they reside in the tunnel barrier or on the surface of the superconductor.^19−21^ We notice that the tunnel junctions with a large overlap with the edge of the NbSe_2_ crystal exhibit multiple features in d*I*/d*V* both outside and inside the gap (Figure 2d). The repeatability of these features in multiple sweeps indicates that they represent discrete energy levels and do not arise from time-dependent noise. They likely arise from defects present at the NbSe_2_ edge cleaved during exfoliation, some of which strongly couple to the superconductor and show up as subgap excitations. The features outside the gap may be understood as resonant features arising in the normal-dot-normal system. While it may not be surprising that the NbSe_2_ edge hosts many defects, this may be critical for the topological edge states predicted in NbSe_2_ with an applied in-plane magnetic field.^5,6^ Instead, it would be crucial to engineer the boundary of the topological and the trivial phase on the bulk of NbSe_2_, as in a recent study.^34^

In a simple planar tunnel junction, a part of the normal “wire” always crosses the NbSe_2_ edge (Figure 2d schematic). Therefore, next we address whether all of the subgap states that we observe arise from such defect states at the edge of NbSe_2_. We do this by electrically isolating the NbSe_2_ edge by transferring additional MoS_2_ layers over the edge of NbSe_2_; see the optical image and the schematic in Figure 2e. The corresponding d*I*/d*V* curves plotted in Figure 2e exhibit a well-behaved superconducting gap. The subgap states are now rare but still present in multiple junctions, as shown in the inset. This points to other sources of defect states, in addition to those at the edge of the NbSe_2_ crystal. The possibilities that remain are the defect states in the tunnel barrier or on the surface of the superconductor.

To address this, we replace the MoS_2_ tunnel barrier with three layers of hBN, known to be an effective tunnel barrier. In particular, the defect density in hBN is small^22,35^ and likely 3 orders of magnitude smaller than that in MoS_2_,^36−39^ although we are not aware of direct comparative studies. The differential conductance for six such tunnel junctions, each with an area of ∼10 μm^2^, is shown in Figure 2f. While tunnel spectroscopy shows a well-behaved superconducting gap with a suppression factor of *G*_N_/*G*_0_ ≈ 300, we do not observe subgap features in any hBN tunnel junction down to our measurement resolution, as evident from the log-scale plot in the inset of Figure 2f. This leads us to believe that the subgap features in MoS_2_/NbSe_2_ tunnel junctions arise either from the edge of the NbSe_2_ crystal or from defects in MoS_2_ that strongly couple to the superconductor.

Furthermore, we study the subgap excitation spectrum in an applied in-plane magnetic field. The Zeeman splitting of the doublet states {|↑⟩, |↓⟩} results in unique features in the excitation spectrum which allows the identification of the ground state. One such measurement is shown in Figure 2c, where at *B*_∥_ = 0 two subgap excitations are visible at *V* ≈ ±0.13 mV. With an applied in-plane magnetic field *B*_∥_, the subgap features split (effective *g* factor of ∼0.7), where one branch moves toward zero bias and the other (weakly visible for *V* > 0) moves toward the gap edge. (See the SI for the second derivative.) The overall behavior can be understood by considering that the dot is in a singlet  ground state at *B*_∥_ = 0. At *V* ≈ 0.13 mV, the chemical potential of the normal lead is aligned to the spin-degenerate doublet excited state. With increasing *B*_∥_, the doublet splits, resulting in the excitation energy to the lower branch decreasing while the excitation energy to the upper branch increases, as illustrated in the Figure 2c schematic. In fact, for increasing *B*_∥_ when the lower branch crosses zero energy, the system undergoes a quantum phase transition and the ground state changes to the doublet ground state. See the SI for an example. The appearance of the bound state sticking to zero energy for *B*_∥_ > 6 T is either the result of two wide (fwhm ≈ 0.18 meV) bound states crossing or the influence of spin–orbit mixing with higher orbital levels, as discussed later.

The ground state of the dot coupled to a superconductor depends on the relative strengths of various energy scales: the tunnel coupling of the dot to the superconductor Γ_s_, the charging energy *U*, the superconducting gap Δ, and the energy of the dot level relative to the chemical potential of the superconductor ξ_d_. Since a finite Γ_s_ is necessary for the visibility of the subgap excitations and a large Γ_s_ favors a singlet ground state, we observe singlet states nearly 6 times as frequently as doublet ground states. (See the SI for a count of ground states.) One such case is shown in Figure 3a, where the excitations at ∼±0.08 meV (*B*_∥_ = 0) move to higher absolute energies with an applied *B*_∥_, as expected for a doublet ground state. The schematic in Figure 3a demonstrates the mechanism. The subgap excitation visible at higher energies of ∼±0.25 meV (*B*_∥_ = 0) may be attributed to the transition to the higher singlet, but this is unlikely due to a different *g* factor. Instead, this may result from another parallel Andreev bound state formed via a second defect, in a junction of size ∼3.5 μm^2^, and a large SOC may result in a nearly vanishing *g* factor as discussed later.

**Figure 3:**
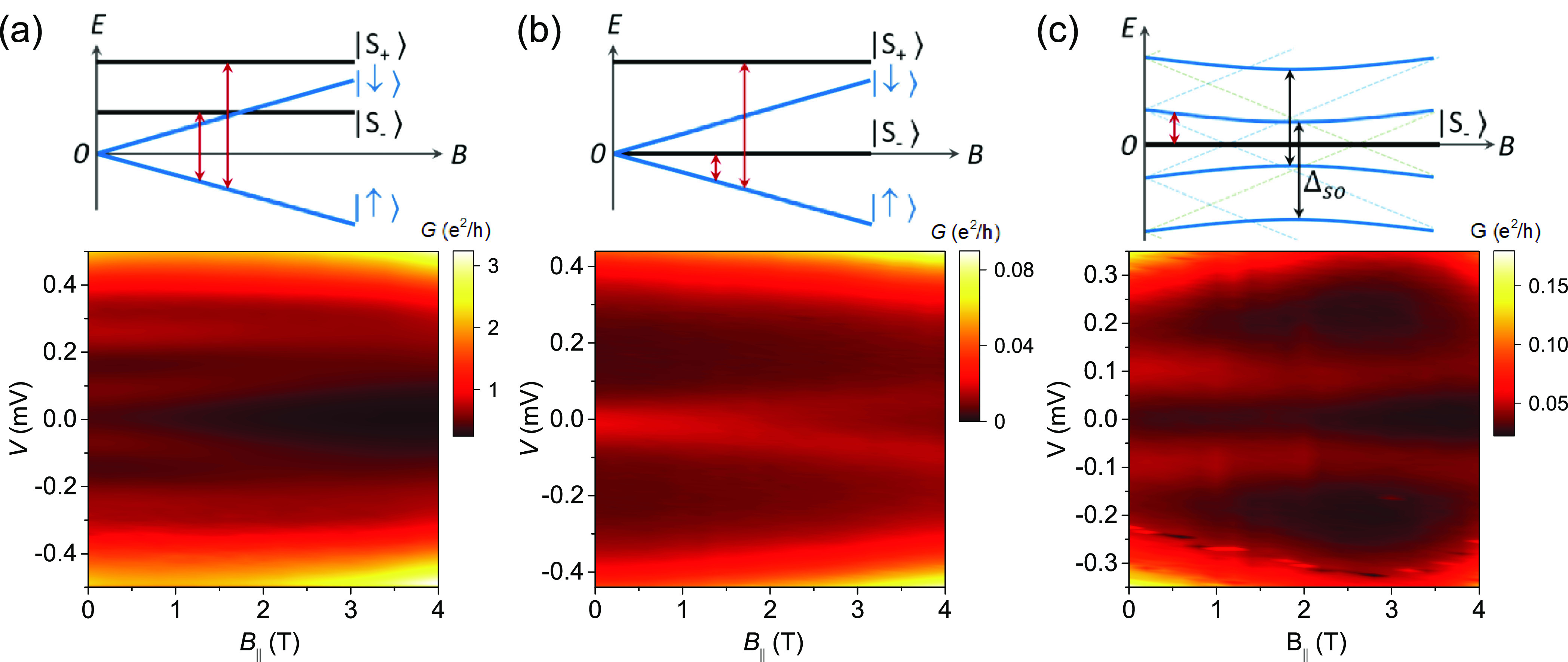
Anomalous subgap excitations. (a) Multiple subgap states are seen at *B* = 0. They evolve with an applied in-plane magnetic field but with a different *g* factor. (b) d*I*/d*V* measurements show a zero bias excitation at *B* = 0, which splits in the magnetic field. The transition to the higher singlet is likely at the gap edge and is not visible in our measurement. (c) Subgap excitations show an avoided crossing feature with a minimal splitting of ∼0.185 meV at *B*_∥_ ≈ 2 T.

For some junctions, a zero-bias peak is also observed at *B*_∥_ = 0 which splits for finite *B*_∥_, as shown in Figure 3b. We believe this results from an accidental degeneracy of the doublet and the lower singlet , as shown in the Figure 3b schematic. Such a spectrum could also result from a doublet ground state where inelastic co-tunneling transitions occur between the two states of the spinful doublet. However, we believe that the transfer of charge through the parity-conserving doublet is strongly suppressed due to (a) the hard gap that we observe and (b) the consequently fourth-order nature of this process. We therefore think that we do not observe them in any of our experiments.

Finally, an avoided-crossing-like feature is shown in Figure 3c, where the subgap excitations move toward zero bias but at *B*_∥_ ≈ 2 T they start to move to higher absolute energies. We attribute this to the spin mixing and hybridization of the doublet states that arise from higher orbital levels, due to SOC in the host material,^40^ as illustrated in the Figure 3c schematic. The size of the splitting depends on the details of the defect which determine the strength of SOC and the relative directions of *B*_SO_ and *B*_∥_. No hybridization occurs when the externally applied magnetic field is parallel to the internal spin orbit field.^41,42^ This may explain why splitting is not observed in other junctions. A large spin–orbit gap (compared to the doublet excitation energy) would also result in a reduced effective *g* factor (see also Figure 3a).

In conclusion, we have performed tunnel spectroscopy on NbSe_2_ by utilizing MoS_2_ or hexagonal boron nitride (hBN) as a tunnel barrier and Ti/Au as the normal leads. We find that the single-particle gapped spectrum often exhibits the presence of subgap excitations, and we probe their origin by studying various heterostructure designs. We show that the edge of NbSe_2_ hosts many defect states, some of which are strongly coupled to the superconductor. However, we also observe subgap excitations in devices where the NbSe_2_ edge is electrically isolated. We show that while the subgap excitations are fairly ubiquitous in MoS_2_ tunnel barriers they are absent in hBN tunnel barriers, suggesting that these subgap excitations arise from the defects in MoS_2_. The evolution of subgap excitations in an applied in-plane magnetic field allows us to probe the magnetic nature of the participating subgap states and reveals the nature of subgap ground states. Subgap excitations that anticross or show no dispersion with the Zeeman field highlight the role of spin–orbit coupling in the system.

## Data Availability

All data in this publication are available in numerical form at https://doi.org/10.5281/zenodo.6817129.

## Supporting Information Available

Detailed descriptions of device fabrication, experimental methods, and complementary measurements (PDF)
