# Supplementary material for: Origin of Subgap States in Normal-Insulator-Superconductor van der Waals Heterostructures
Source: Nano Lett. 2023 Mar 16;23(7):2454–9. doi: 10.1021/acs.nanolett.2c02777 (PMC10103330; doi:10.1021/acs.nanolett.2c02777)
Supplement: Supplementary file 1 — nl2c02777_si_001.pdf [file nl2c02777_si_001.pdf]

Supporting Information: Origin of subgap states in normal-insulator-superconductor van der Waals heterostructures

Paritosh Karnatak,[1] Zarina Mingazheva,[1] Kenji Watanabe,[2] Takashi Taniguchi,[3] Helmuth Berger,[4] László Forró,[4,5] and Christian Schönenberger[1,6]

[1]*Department of Physics, University of Basel, CH-4056 Basel, Switzerland*

[2]*Research Center for Functional Materials, National Institute for Material Science, 1-1 Namiki, Tsukuba 305-0044, Japan*

[3]*International Center for Materials Nanoarchitectonics, National Institute for Materials Science, 1-1 Namiki, Tsukuba 305-0044, Japan*

[4]*Institute of Condensed Matter Physics, Ecole Polytechnique Fédérale de Lausanne, CH-1015 Lausanne, Switzerland*

[5]*Stavropoulos Center for Complex Quantum Matter, Department of Physics, University of Notre Dame, Notre Dame, IN 46556, USA*

[6]*Swiss Nanoscience Institute, University of Basel, CH-4056 Basel, Switzerland*

I. FABRICATION AND MEASUREMENT

The normal-insulator-superconductor (NIS) type planar tunnel junctions are fabricated by dry stacking of MoS_2 ($3-5$ layers) or hBN (3 layers) on $NbSe_2$ crystals (~ 3 nm -20 nm). Polycarbonate (PC) backed by PDMS and glass is used for the standard pickup method. The stacking is performed in a glovebox in N_2 atmosphere with H_2O and O_2 levels below 1 ppm. PC is dissolved in dichloromethane. While MoS_2 or hBN act as the tunnel barrier as well as prevent $NbSe_2$ from oxidation during device processing the exposure to the ambient is minimised during device processing. Moreover we encapsulate thin $NbSe_2$ (~ 3 nm) from both sides to prevent degradation. Nearly all devices also contain a graphite crystal transferred over $NbSe_2$, which acts as an ohmic contact to $NbSe_2$. Normal contact regions are defined by ebeam lithography and Ti/Au (5 nm/50 nm) is deposited by ebeam evaporation to create the tunnel junctions. The area of the tunnel junctions is typically ~ 1 μm^2 to 3 μm^2, unless stated otherwise. All details of device parameters can be found in the device table at the end of the Supporting Information (SI).

The measurements were performed in a ^{3}He fridge at a base temperature of ~ 250 mK. The electrical lines are filtered by using pi-filters at the breakout box and tapeworm filters at the cold finger. A small ac voltage ($V_{ac} < k_BT$, where k_B is the Boltzmann constant and T is the cryostat temperature) is added to the biasing dc voltage V using a transformer and the lockin amplifier

records the output current via an external current-voltage amplifier. Due to a cryostat installation error the actual magnetic fields may be smaller by up to 10 % than reported here.

II. TUNNEL SPECTRA FITTING

In the main text we presented a fit of the tunnel spectra with simple BCS-type density of states, shown in Figure 1b also reproduced here in Figure S1a. However, the tunnel spectra of $NbSe_2$ is known to exhibit features that deviate from the simple BCS model (an isotropic, single gap). Other models have been employed to fit the tunnel spectra, and two important ones are 1) The two-band model [1, 2], for which a fit is shown in Figure S1b and 2) the anisotropic-gap (single gap) model [3] with a fit in Figure S1c. The temperature for all fits is fixed to the measurement temperature, $T = 255$ mK. In our view the two band model presents the most consistent picture, where the superconductivity in thicker $NbSe_2$ is thought to arise from multiple bands. With the layer number decreasing in $NbSe_2$ the superconducting gap approaches a single gap model and is consistent with bandstructure calculations. In contrast it is hard to justify the change of anisotropy as the $NbSe_2$ becomes thinner.

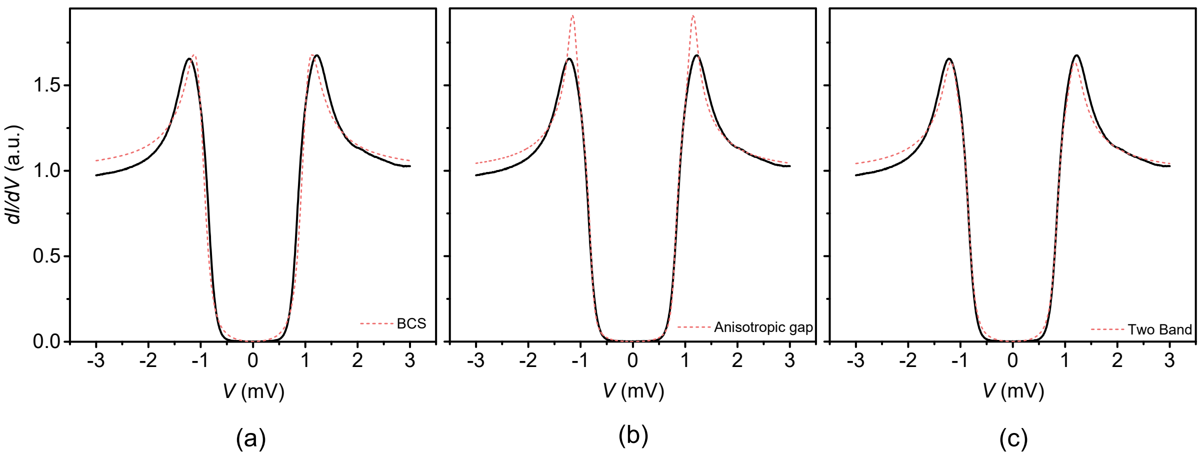

FIG. S1: Tunnel spectra fitting. (a) A single gap BCS model with parameters $\Delta = 1.0$ meV, $\Gamma = 0.11$ meV. (b) A single anisotropic gap with $\Delta = 1.14$ meV, $\Gamma = 0.045$ meV and $A = 0.15$. (c) Two band model with parameters $\Delta_1 = 1.1$ meV, $\Gamma_1 = 0.08$ meV, $\Delta_2 = 0.9$ meV, $\Gamma_2 = 0.08$ meV and $N_1/N_2 = 0.6$.

III. TEMPERATURE DEPENDENCE OF THE TUNNEL SPECTRA

Here we highlight the role of superconductivity by showing temperature dependent tunnel spectra. The tunnel spectra exhibits broadening with temperature, as shown in the differential conductance map in Figure S2a. The superconducting gap is no longer visible for $T \geq 4.4$ K and is reasonable for a thin (~ 3 nm) $NbSe_2$. In addition, the subgap excitations visible at a base temperature $T = 270$ mK are no longer visible at $T = 1.5$ K, as shown in Figure S2b. Similarly, for another device D21, shown in Figure S2c, the subgap excitations are no longer visible at a larger temperature $T = 4$ K, and the tunnel spectrum exhibits thermal broadening.

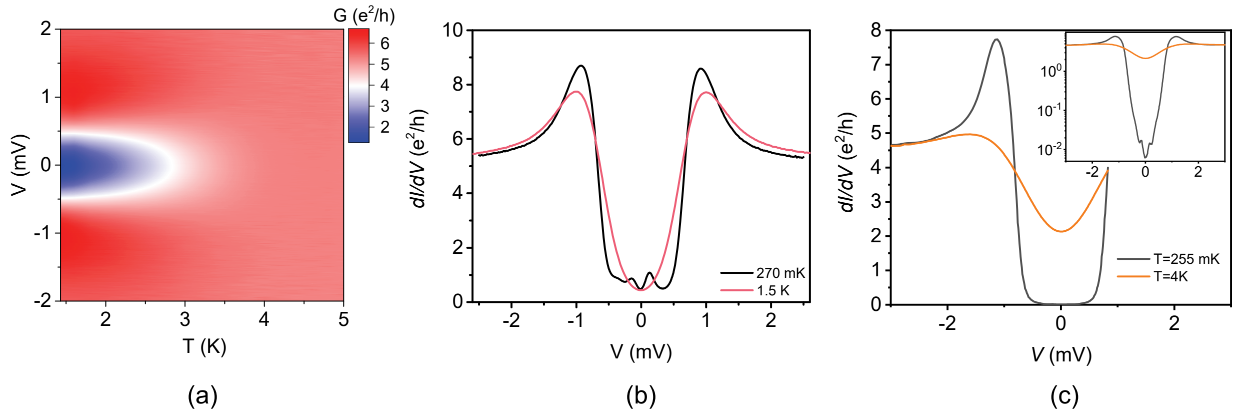

FIG. S2: Temperature dependence of the tunnel spectra. (a) Differential conductance for device D10 as a function of voltage bias V and temperature T, starting at $T = 1.43$ K. (b) Differential conductance measurements for device D10 show that the thermal broadening at $T = 1.5$ K is large enough that the subgap excitations are not visible when compared to $T = 270$ mK. (c) Differential conductance measurements for device D21 at $T = 270$ mK and $T = 4$ K.

IV. SECOND DERIVATIVE

Figure S3 demonstrates the upper branch of the doublet presented in Figure 2(c) of the main text. For this, the second derivative of the differential conductance with respect to the bias voltage was taken. In the main text we indicate bias voltage as V, here, bias voltage is indicated both as V_b and V.

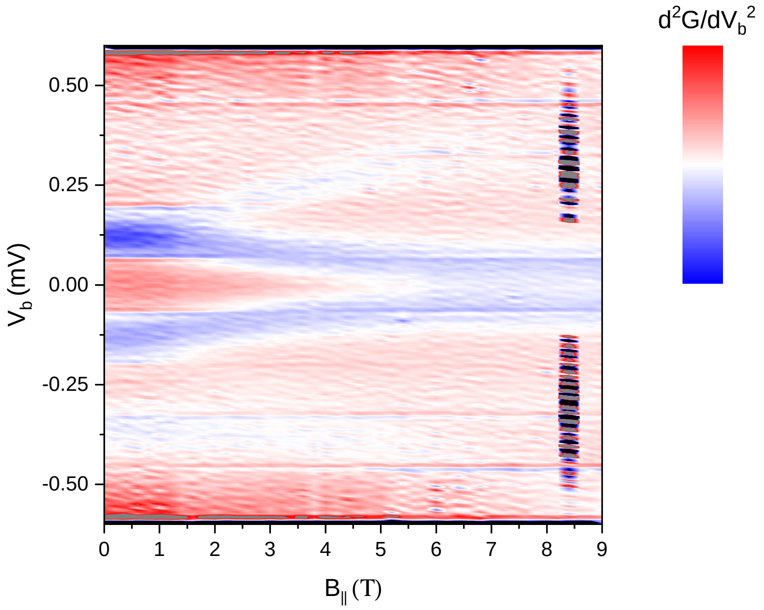

FIG. S3: 2nd derivative of the differential conductance with respect to the bias voltage. Here G is normalized conductance. Besides branches of doublets which are possible to see in the main text, the second derivative shows clearer the upper branch of the doublet at the positive bias voltage.

V. ADDITIONAL SUBGAP SPECTRA 1

While measuring in the wide range of magnetic field (Figure S4 and Figure S5), we take care of conductance jumps due to vortices by shifting dI/dV. The shift was done such that at all magnetic field values, the conductance at $V_b = 0$ has the same value.

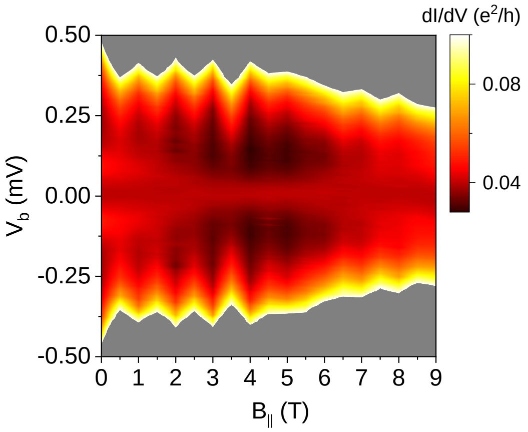

(a)

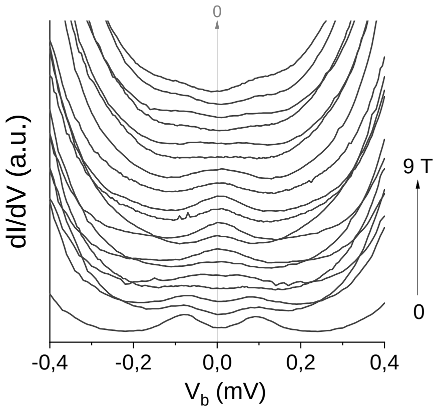

(b)

FIG. S4: Magnetic field evolution of the subgap excitations. This is another example of the transition of the ground state from singlet to doublet under in-plane magnetic field measured for the device D21, tunnel junction no.10. (a) Colour map of dI/dV as a function of in-plane magnetic field $B_{\|}$ and bias voltage V_b. (b) Shifted differential conductance curves for the same tunnel junction as in (a). The magnetic field step size of the curves is 0.5 T.

VI. ADDITIONAL SUBGAP SPECTRA 2

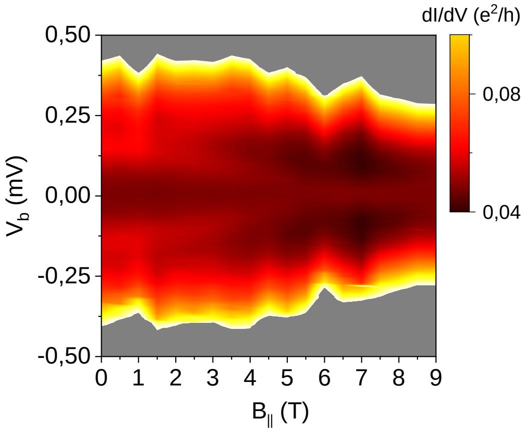

(a)

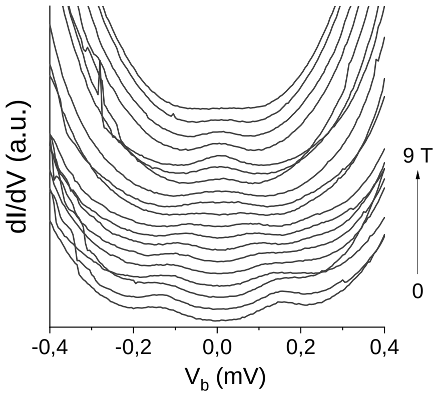

(b)

FIG. S5: Magnetic field evolution of the subgap excitations, device D21, tunnel junction no.13. (a) Colour map of dI/dV as a function of in-plane magnetic field $B_{\parallel}$ and bias voltage V_b. (b) Shifted differential conductance curves for the same tunnel junction as in (a). The magnetic field step size of the curves is 0.5 T. At $B_{\parallel} = 9$ T, the full width at half maximum (FWHM) is around 0.5 meV. Such a wide peak hinder us to distinguish if the lines cross as in Figure S4a or if the dI/dV peak sticks to V_b=0 above 6 T.

VII. DEVICE DETAILS

The table in this section lists the details of all measured tunnel junctions, particularly geometrical parameters and the suppression factor of the superconducting gap G_N/G_0. In the column "Figure", we indicated in what figures the junction was presented. The main focus is on the type of ground state and its evolution under an in-plane magnetic field (g-factor).

The cases of the singlet ground state, a doublet ground state and accidental degeneracy are shown in the main text in Figure 2c, Figure 3a and Figure 3b respectively. The tunnel junctions for which either the magnetic dependence of the subgap excitations was not studied or the ground state is ambiguous, as in Figure S6a have been categorised as 'Unknown' (ground state). The tunnel junctions for which subgap excitations were not visible, as in Figure S6b have been categorised as 'None'.

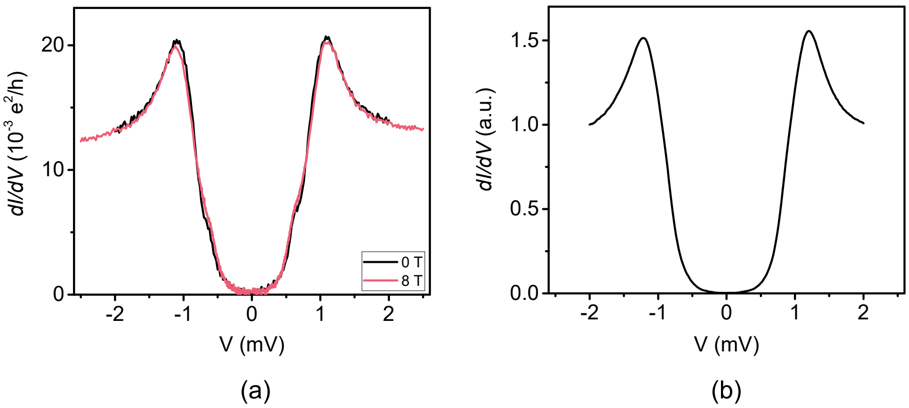

FIG. S6: Additional definitions for the subgap ground state categories. (a) Tunnel spectra when the subgap ground state cannot be determined are categorised as 'Unknown'. Shown is an example of the subgap features that have a vanishing g-factor for junction 15 device D10. (b) Tunnel spectra where the subgap features are not visible are categorised as 'None' for junction 17 device D10.

A count of the ground states shown in Figure S7 summarized our measurements. More than half of tunnel junctions show subgap excitations, however many of them do not show clear behaviour under an in-plane magnetic field or suppress under a small magnetic field. From the behaviour of the rest of the tunnel junctions, we believe six tunnel junctions have singlet ground state, one - doublet, and four tunnel junctions show accidental degeneracy of a doublet and a hybridized singlet states.

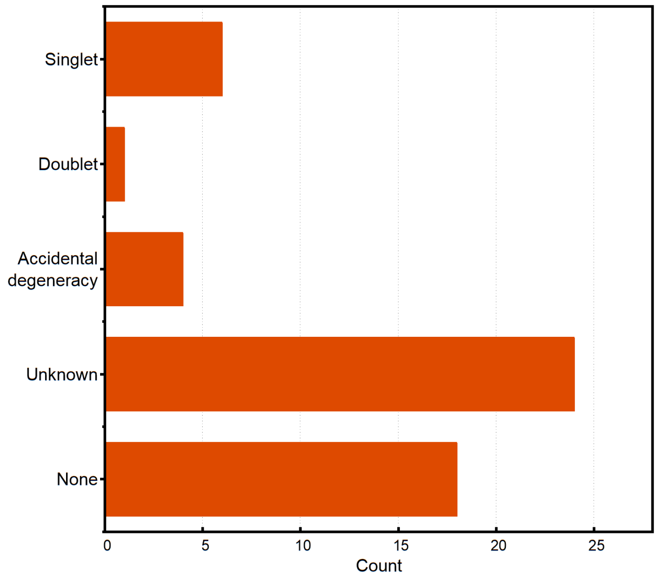

FIG. S7: Count of ground states of observed in-gap Andreev bound states

REFERENCES

[1] Dvir, T., Massee, F., Attias, L. et al. Spectroscopy of bulk and few-layer superconducting $NbSe_2$ with van der Waals tunnel junctions. Nat. Commun. 9, 598 (2018).

[2] Noat, Y., Silva-Guillén, J. A., Cren, T. et al. Quasiparticle spectra of $2H-NbSe_2$: Two-band superconductivity and the role of tunneling selectivity. Phys. Rev. B 92, 134510 (2015)

[3] Khestanova, E., Birkbeck, J., Zhu, M. et al. Unusual Suppression of the Superconducting Energy Gap and Critical Temperature in Atomically Thin $NbSe_2$. Nano Letters 18, 2623-2629 (2018)

Details of all measured tunnel junctions (53 junctions)									
**Device**	**Junction**	**$NbSe_2$ thickness, nm**	**Tunnel barrier, layers (L)**	**Area of Tj um^2**	**G_N/G_0**	**Ground state**	**g-factor**	**Comments**	**Figure**
D10	10	3	3L MoS_2	3.2	11	Singlet	0.70		2a, 2c, S3
	15	3	4L MoS_2	1.1	49	Unknown	0.00	Subgap states at the gap edge	2a, S6(a)
	16	7	2L MoS_2	2.6	4	Singlet	0.70	Approximate g factor	2a
	17	7	4L MoS_2	2.7	400	None			2a, S6(b)
D14	1	12	4L MoS_2	4	63	Unknown		on $NbSe_2$ edge	
	5	12	4L MoS_2	2.4	328	Singlet	0.80	Anticrossing like feature	3c
	7	12	4L MoS_2	3.5	100	Doublet	0.64	Higher excitation has a vanishing g-factor	3a
	14	5	3L MoS_2	1.3	1	Unknown		On $NbSe_2$ edge	
	15	10	3L MoS_2	2.3	24	Unknown			
	16	10	3L MoS_2	2.4	4	Unknown			
	17	10	3L MoS_2	4.1	3	Accidental degeneracy	1.70		
	18	10	3L MoS_2	4.2	4	Unknown	0.00		
	19	10	3L MoS_2	1.3	44	Unknown			
D19	3	7	3L hBN	10	826	None			2f black
	5	7	3L hBN	9.8	807	None			2f red
	7	7	3L hBN	10	1563	None			2f blue
	10	7	3L hBN	10	571	None			2f green
	11	7	3L hBN	10	760	None			2f violet
	16	7	3L hBN	12.2	500	None			2f yellow
D20	1	20	3L MoS_2	2.7	300	Unknown		$NbSe_2$ edge isolated by MoS_2	
	2	20	3L MoS_2	2.7	150	Unknown		$NbSe_2$ edge isolated by MoS_2	2e black
	3	20	3L MoS_2	2.3	167	Unknown		$NbSe_2$ edge isolated by MoS_2	
	4	20	3L MoS_2	2.5	80	Accidental degeneracy	0.55	$NbSe_2$ edge isolated by MoS_2	2e red

Device	Junction	$NbSe_2$ thickness, nm	Tunnel barrier, layers (L)	Area of Tj um^2	G_N/G_0	Ground state	g-factor	Comments	Figure
D20	5	20	3L MoS_2	2.3	10	None			
	6	20	3L MoS_2	3.1	27	Singlet	0.45	Doublet upper branch not visible	
	7	20	3L MoS_2	3	242	None			
	8	20	3L MoS_2	2.9	80	None			
	9	10	3L MoS_2	1.8	131	None			
	10	10	3L MoS_2	1.8	410	None			
	11	10	3L MoS_2	3.2	141	None			
	12	10	3L MoS_2	1.8	8	None		V-shaped gap	
	13	10	3L MoS_2	2.5	2	Unknown		V-shaped gap	
	14	10	3L MoS_2	2.9	104	None			
	16	10	3L MoS_2	3.5	344	None			
D21	10	8	4L MoS_2	1.52	408	Singlet	0.75		1d, S4
	13	6	4L MoS_2	1.1	425	Singlet	0.67		S5
	12	8	4L MoS_2	0.71	150	Accidental degeneracy	0.64	On $NbSe_2$ edge	3b
	6	8	4L MoS_2	1.96	554	Accidental degeneracy			
	7	8	4L MoS_2	1.6	587	Unknown			S2(c)
	9	8	4L MoS_2	1.96	303	Unknown			
	16	6	4L MoS_2	1.25	630	None			
	11	8	4L MoS_2	1.56	650	Unknown			
	20	6	4L MoS_2	1.4	920	Unknown			
	14	6	4L MoS_2	1.49	271	None			
MN1	7	11	3L MoS_2	1.56	826	Unknown		Partly located on the hole of MoS_2	1b, 1c, S1
	17	11	3L MoS_2	0.68	6	Unknown		On $NbSe_2$ edge	2d blue
MN4	16	8	5L MoS_2	3	1013	Unknown			
	15	8	5L MoS_2	8	54	Unknown		on $NbSe_2$ crystal step	
	14	8	4L MoS_2	9.1	334	Unknown		on $NbSe_2$ crystal step	

**Device**	**Junction**	**$NbSe_2$ thickness, nm**	**Tunnel barrier, layers (L)**	**Area of Tj um^2**	**G_N/G_0**	**Ground state**	**g-factor**	**Comments**	**Figure**
MN4	11	10	4L MoS_2	4.6	359	Unknown			
	3	14	5L MoS_2	3.5	327	Unknown		on $NbSe_2$ edge	
	9	10	5L MoS_2	6.2	52	Unknown		on $NbSe_2$ edge	2d black and red
	17	8	5L MoS_2	3.64	145	Unknown		Subgap states at the gap edge	
